# Supplementary material for: Interpretable brain age prediction using linear latent variable models of functional connectivity
Source: PLoS One. 2020 Jun 10;15(6):e0232296. doi: 10.1371/journal.pone.0232296 (PMC7286502; doi:10.1371/journal.pone.0232296)
Supplement: S1 Appendix — (PDF) [file pone.0232296.s001.pdf]

S1 Appendix. Technical details of the MHA algorithm. In this appendix, we give further details of the block-coordinate descent algorithm which we implement to update the model parameters. In practice, we solve the constrained optimisation (8) via the use of projections onto the non-negative quadrant (non-negativity) and Lagrange multipliers. More specifically, we use the objective function:

$$\tilde{\mathcal{L}} = \mathcal{L} + \frac{\delta}{2}||W^T W - I_k||_2^2 + \mathrm{tr}(\Gamma^T(W^T W - I_k)), \tag{16}$$

where $\Gamma \in \mathbb{R}^{k \times k}$ and δ are Lagrange multipliers enforcing the orthonormality constraints.

We employ gradient descent approach to update the estimate of W. To this end, we follow Monti and Hyvärinen [29] and introduce a gradient step size η and project onto the non-negative orthant at each iteration (this ensures that the positivity constraint is maintained). The update takes the form

$$W \leftarrow \mathcal{P}^+ \left(W - \eta \left(\frac{\partial \mathcal{L}}{\partial W} + \delta(WW^\top W - W) + W\Gamma) \right) \right), \tag{17}$$

where $\mathcal{P}^+ = \max(0, x)$ denotes the projection onto the non-negative orthant and η is a stepsize parameter. The update for the Lagrange multipliers Γ is given by:

$$\Gamma \leftarrow \Gamma + \delta(W^\top W - I).$$

In the case of the loading matrix, the gradient update is defined as:

$$\begin{aligned} \frac{\partial \mathcal{L}}{\partial W} &= \sum_{i=1}^{N} \frac{\partial \mathcal{L}}{\partial \Sigma^{(i)}} \frac{\partial \Sigma^{(i)}}{\partial W} \\ &= \sum_{i=1}^{N} \left(-{\Sigma^{(i)}}^{-1} + {\Sigma^{(i)}}^{-1} S^{(i)} {\Sigma^{(i)}}^{-1} \right) W G^{(i)} \ , \end{aligned} \tag{18}$$

where we note that via the Sherman-Woodbury identity and using the form of the covariance (3), we can write ${\Sigma^{(i)}}^{-1}$ as follows:

$${\Sigma^{(i)}}^{-1} = {(v^{(i)}I)}^{-1} - {(v^{(i)}I)}^{-1} W({G^{(i)}}^{-1} + W^\top v^{(i)} I W)^{-1} W^\top {(v^{(i)}I)}^{-1}. \tag{19}$$

For the diagonal matrix of eigenvalues, $G^{(i)}$, we can update each matrix independently as follows:

$$\begin{aligned} \frac{\partial \mathcal{L}}{\partial G^{(i)}} &= \frac{\partial \mathcal{L}}{\partial \Sigma^{(i)}} \frac{\partial \Sigma^{(i)}}{\partial G^{(i)}} \\ &= \sum_{i=1}^{N} \left(-{\Sigma^{(i)}}^{-1} + {\Sigma^{(i)}}^{-1} K^{(i)} {\Sigma^{(i)}}^{-1} \right) \ . \end{aligned} \tag{20}$$
