# Supplementary material for: Interpretable brain age prediction using linear latent variable models of functional connectivity
Source: PLoS One. 2020 Jun 10;15(6):e0232296. doi: 10.1371/journal.pone.0232296 (PMC7286502; doi:10.1371/journal.pone.0232296)
Supplement: S1 Code — (PDF) [file pone.0232296.s002.pdf]

S2 Code. Python and R implementations of the MHA algorithm.

- Python: https://github.com/piomonti/MHA
- R: http://www.gatsby.ucl.ac.uk/~ricardom/FactorCovariance_ScoreMatch_PenalizeLagrange.R
