# Supplementary material for: Interpretable brain age prediction using linear latent variable models of functional connectivity
Source: PLoS One. 2020 Jun 10;15(6):e0232296. doi: 10.1371/journal.pone.0232296 (PMC7286502; doi:10.1371/journal.pone.0232296)
Supplement: S1 Table — In the case of the HCP datasets, 80 subjects were randomly selected out of all possible subjects. (PDF) [file pone.0232296.s003.pdf]

Dataset	# subjects	Age range
CamCAN	647	18—88
HCP	80	20—36
ATR	191	20—70

Table 1. Table detailing number of subjects studied in each of the three datasets considered. In the case of the HCP datasets, 80 subjects were randomly selected out of all possible subjects.
