# Supplementary figures and images for: Interpretable brain age prediction using linear latent variable models of functional connectivity
Source: PLoS One. 2020 Jun 10;15(6):e0232296. doi: 10.1371/journal.pone.0232296 (PMC7286502; doi:10.1371/journal.pone.0232296)

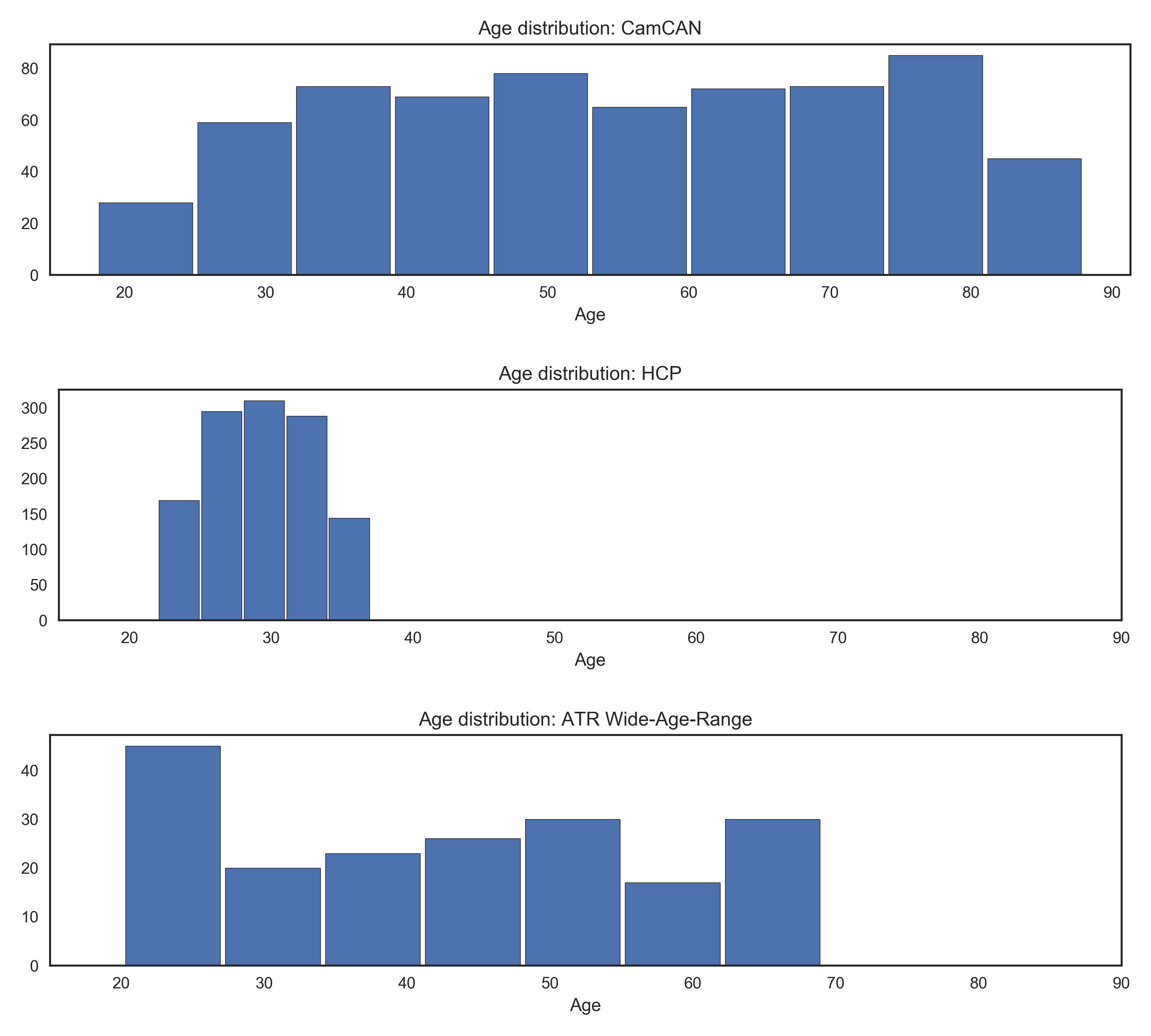

Supplement: S1 Fig — (PNG) [file pone.0232296.s004.png]

# PCA

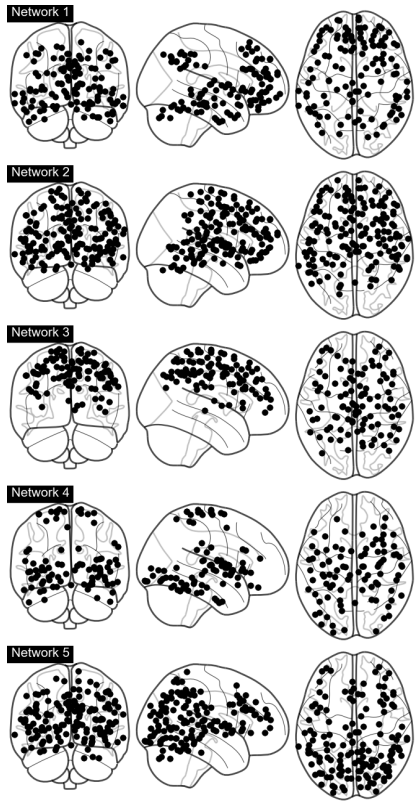

# Factor Analysis

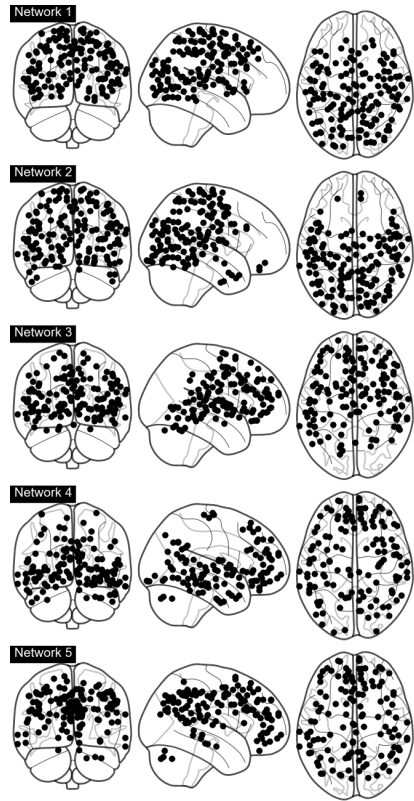

# MCF

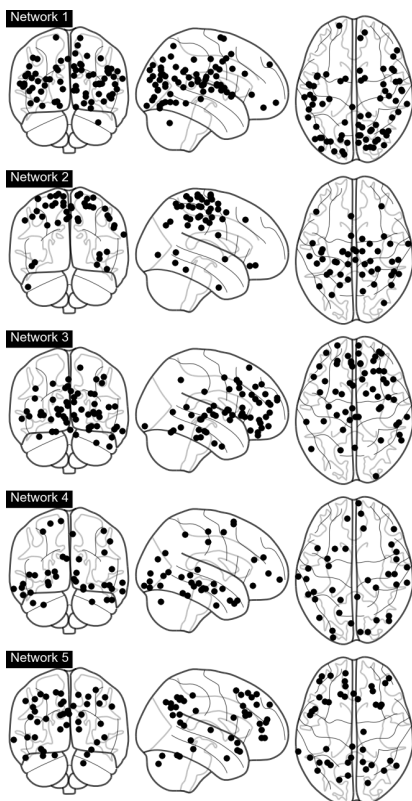

# Non-neg PCA

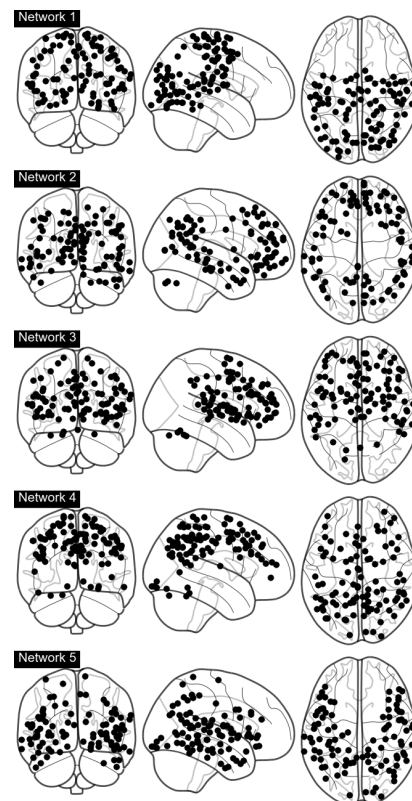

# ICA

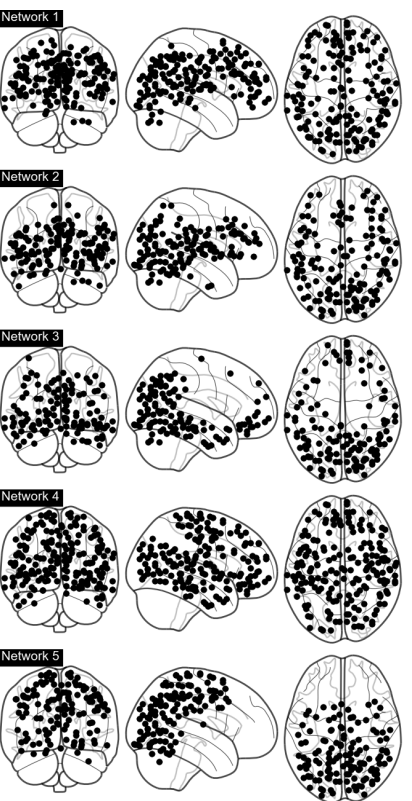

Supplement: S2 Fig — (PARTIAL) [file pone.0232296.s005.partial]

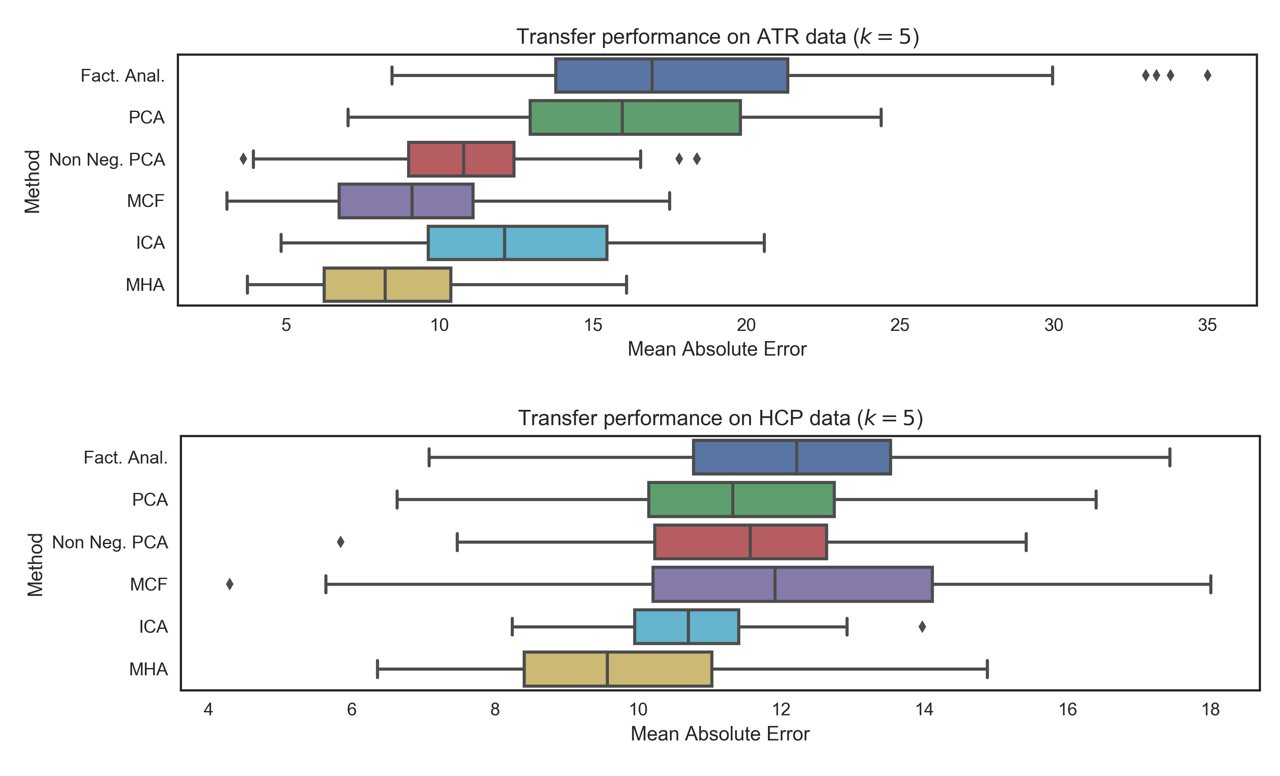

Supplement: S3 Fig — (PNG) [file pone.0232296.s006.png]
