# Supplementary material for: Interpretable brain age prediction using linear latent variable models of functional connectivity
Source: PLoS One. 2020 Jun 10;15(6):e0232296. doi: 10.1371/journal.pone.0232296 (PMC7286502; doi:10.1371/journal.pone.0232296)
Supplement: Attachment — Submitted filename: ReplyReviewers.pdf [file pone.0232296.s007.pdf]

Reply to Reviewers: PLOS Submission PONE-D-19-33576

February 27, 2020

We thank the editor and reviewers for providing helpful feedback and suggestions. These suggestions have been incorporated into an updated manuscript, significantly improving the quality of our submission. Below we reply to each of the reviewers comments and outline the changes introduced in the updated manuscript (these are also highlighted in blue for clarity).

Reply to Reviewer 1

- *The work proposed a very interesting a powerful analytical framework to study the aging dynamics of human functional connectivity. The mathematical presentation is flawless and clearly presented.*

  We thank the reviewer for his encouraging words.

- *Interestingly, the method appeared extendable to different contexts studying the dynamical aspects of functional connectivity, a relevant topic to date. First of all, authors should consider this aspect in the discussion/conclusion section.*

  We thank the reviewer for highlighting this important further extension of our method. Following from the reviewers comments, we have added the following sentences to the final paragraph of the conclusion (lines 601—607 of the updated manuscript):

  > Moreover, an avenue for further research would be to consider performing classification instead of regression in the second stage of the proposed method. Whilst a natural task would be to discriminate between healthy controls and subjects with some neuropathology, such an approach could also be employed in the context of task-based fMRI as well as to study changes in functional connectivity induced by various distinct tasks (Zippo et al., 2019a) or neuropathologies (Lorenz et al., 2018; Zippo et al., 2019b).

- *Although authors use HCP Young Adult dataset just for test, they should declare the number of subject used.*

We thank the reviewer for bringing this to our attention. We have now added a table in the Supplementary Material (page 22 of the updated manuscript) clearly detailing the number of subjects considered for each dataset and the relevant age ranges.

- *Most importantly, within the human connectome project, there exists a similar collection called "HCP Aging" chracterized by 1200 Subjects in the age range of 36-100+ years old. That's the dataset they should test.*

  We agree with the reviewer that the HCP Aging dataset is an exciting dataset to study. However, to date only 129 of the total 1200 subjects have been processed for this dataset[1]. As such, it is currently a smaller dataset than two of the three open-access datasets we consider: CamCAN has 647 subjects whilst the ATR Wide-Age-Range dataset has 191. As such, we believe this dataset should be left to future work when the entirety of the 1200 subjects are available for study.

- *If, the python* `plot_glass_brain` *function has been used to plot figures 5 and S4 (as I assumed), they should state it because otherwise it is necessary to specify the x-y-z coordinates. That function put in foreground every network elements (nodes/edges) and the brain in background and it is particularly useful in displaying brain network.*

  We thank the reviewer for highlighting this omission. We did indeed use the `plot_glass_brain` function. We have added a footnote on line 412 of the updated manuscript to reflect this.

- *However, authors stated (in captions and text) those plots as "networks" but just nodes (ROI centroids?) are presented. This discrepancy should be fixed.*

  We thank the reviewer for altering us to this issue. The plots shown in Figure 5 reflect the inferred sub-networks based on the MHA algorithm discussed in Section 2.1. As such, all regions within a given sub-network share strong positive correlations, which we omit from Figure 5 for clarity. The main purpose of this figure is to highlight that the inferred sub-networks correspond to spatially consistent brain regions.

  > We note that, as discussed in Section 2.1, the MHA linear latent variable model effectively clusters regions into sub-networks via the introduction of non-negativity and orthonormality constraints. As such, each plot in the left panel of Figure 5 visualizes spatially remote brain regions which have been clustered together, indicating that these regions share strong positive correlations. We note that these correlations (i.e., edges in a network) are omitted for clarity in Figure 5.

[1] Latest release was on 05/21/2019, see here for details: `https://www.humanconnectome.org/study/hcp-lifespan-aging`
